# An fMRI study of error monitoring in Montessori and traditionally-schooled children

**DOI:** 10.1038/s41539-020-0069-6

**Published:** 2020-07-17

**Authors:** Solange Denervaud, Eleonora Fornari, Xiao-Fei Yang, Patric Hagmann, Mary Helen Immordino-Yang, David Sander

**Affiliations:** 1 Swiss Center for Affective Sciences (CISA), Campus Biotech, University of Geneva, 1202 Geneva, Switzerland; 2 Connectomics Lab, Department of Radiology, Lausanne University Hospital and University of Lausanne (CHUV-UNIL), 1011 Lausanne, Switzerland; 3 The Laboratory for Investigative Neurophysiology (The LINE), Department of Radiology and Department of Clinical Neurosciences, Lausanne University Hospital and University of Lausanne (CHUV-UNIL), 1011 Lausanne, Switzerland; 4 Biomedical Imaging Center (CIBM), Department of Radiology, Lausanne University Hospital and University of Lausanne (CHUV-UNIL), 1011 Lausanne, Switzerland; 5 Center for Affective Neuroscience, Development, Learning and Education (CANDLE); Brain and Creativity Institute, Rossier School of Education, University of Southern California, Los Angeles, CA 90089 USA; 6 Neuroscience Graduate Program, University of Southern California, Los Angeles, CA USA; 7 Laboratory for the Study of Emotion Elicitation and Expression, Department of Psychology, Faculty of Psychology and Educational Sciences (FPSE), University of Geneva, 1205 Geneva, Switzerland

**Keywords:** Problem solving, Human behaviour

## Abstract

The development of error monitoring is central to learning and academic achievement. However, few studies exist on the neural correlates of children’s error monitoring, and no studies have examined its susceptibility to educational influences. Pedagogical methods differ on how they teach children to learn from errors. Here, 32 students (aged 8–12 years) from high-quality Swiss traditional or Montessori schools performed a math task with feedback during fMRI. Although the groups’ accuracies were similar, Montessori students skipped fewer trials, responded faster and showed more neural activity in right parietal and frontal regions involved in math processing. While traditionally-schooled students showed greater functional connectivity between the ACC, involved in error monitoring, and hippocampus following correct trials, Montessori students showed greater functional connectivity between the ACC and frontal regions following incorrect trials. The findings suggest that pedagogical experience influences the development of error monitoring and its neural correlates, with implications for neurodevelopment and education.

## Introduction

Given the changing landscape of work and the ease of acquiring factual information via technology^1^, there is an active debate around how pedagogical approaches can support students not simply in memorizing facts and becoming proficient at procedures but in developing abilities for evaluating their ongoing learning processes^2^; in essence, for learning how to learn efficiently. Central to this enterprise is fostering a self-directed, process-oriented approach to learning, in which children learn to recognize and utilize information about incorrect responses to iteratively improve their skills^3–6^.

Error monitoring refers to the intrinsic ability to detect and evaluate outcomes that violate expectation and to adapt in response^7^. Existing work suggests that error monitoring shares processing features with surprise or violation of expectations and serves as a basic orienting mechanism for subsequent behavioral adaptation and learning^8–10^. Individuals’ error-monitoring competencies are tightly related to self-regulatory and flexible goal-directed behaviors^7,11^. Along with executive functions, error-monitoring improves across development, with adult-like responses by mid-adolescence^12–14^. Its developmental trajectory is known to depend upon underpinning brain networks, most notably involving the cingulate gyrus, including the anterior cingulate cortex (ACC), and developmental changes in both behavior and brain activity have been described^12,15,16^. Most notably, these include increased capacity to detect errors quickly and self-correct accurately, and corresponding shifts in functional connectivity of the ACC.

It is possible that error monitoring and its neural correlates are shaped by developmental experience, and in particular by schooling. Pedagogical traditions differ in the ways they support children in recognizing and reacting to incorrect responses. While traditional pedagogy typically teaches children by providing them information about when they have made a mistake, feedback that is often delayed^17,18^, so that they can avoid such mistakes in future^19^, Montessori students are typically not given direct information about the correctness of their answers. Instead, Montessori teachers encourage children to notice their own incorrect thinking or to help peers identify incorrect thinking in a pro-social manner^20^. This method was built from the thinking of Maria Montessori in the 1920’s based on her systematic observations of children’s development in educational settings and in interactions^20^. The global aim was to facilitate children actively organizing their own understanding in a socially cooperative context in which children of mixed ages observe and sometimes teach each other^20^. This could implicitly teach Montessori-schooled children to engage with errors and self-correction in a more autonomous, process-oriented and constructive way, while also helping them leverage social skills. This is in contrast to methods that focus children through testing on memory and recall^21,22^, which are emphasized in traditional schooling.

Montessori students have been reported to achieve higher scores on academic tasks^23–25^, on tests of socio-emotional skills^23,26^, and on creativity tests^27,28^. These outcomes are thought to reflect children’s experiences with the pedagogical strategies^29^, but the cognitive origins of these effects have not been studied. Given the focus on independent recognition of errors in Montessori pedagogy, one reasonable hypothesis is that Montessori schooling may be effective in part because it impacts children’s development of error monitoring.

Our previous work supports the hypothesis that error monitoring may differ in children exposed to Montessori versus traditional pedagogy^30^. Compared with Montessori students, traditionally-schooled students were found to react more strongly when detecting an incorrect response, as measured by strength of global field power in EEG, suggesting lower self-regulated error-monitoring ability^31^. These first results suggest that pedagogical practice influences the way young students learn to perceive and respond to errors, with traditional teaching methods potentially teaching children to strive to remember and produce only correct responses^32^. These results align well with research on adults demonstrating that incorrect responses are typically affectively tagged as negative and aversive^33,34^. Of note, the adults participating in the existing studies had likely almost exclusively been traditionally-schooled.

Human studies consistently implicate cingulate regions in general, and the ACC in particular, in the process of error monitoring. This is consistent with functional connectivity and brain activation studies on neurodevelopmental maturation of the cingulate gyrus. These studies report an age-related caudal-ventral gradient of developmental change^35^, that is evident with regard to error monitoring^15^, especially between 8–12 years of age. However, to date, functional neuroimaging studies of error monitoring have mainly been conducted with adults. These studies shed light on the spatio-temporal specificity of error monitoring. They make clear its distinction from conflict responses and reward processes^36^, and highlight its role not only in behavioral change but in social adaptation^8,37,38^. That is, error-monitoring processes are not only invoked for our own mistakes, but also for mistakes that we monitor in others^39–41^. Competitive or cooperative social settings have been shown to differently influence error perception and vicarious learning, such that cooperative settings heighten error-monitoring responses to others’ mistakes, and increase subsequent learning^41^.

Here, we asked 8–12-year-old students from Swiss traditional and Montessori schools to judge whether solutions to straightforward math problems were right or wrong during fMRI scanning, and studied their brain activation patterns when their responses were correct or incorrect using blood oxygen-level dependent (BOLD) activation and functional connectivity analyses. Public traditional and private Montessori schools in Switzerland both provide high-quality education, but differ systematically in their pedagogical approaches (according to local educational policies and the Montessori application standards). To control as best as possible for selection bias, we collected information on demographic factors, families’ reported educational practices and beliefs, as well as on fluid intelligence and math ability, and found students in the two pedagogical groups to be comparable (Table 1).

**Table 1:** Demographic and control variables for the Montessori (M) and traditionally-schooled (T) groups.

	Group				
	M	T	*X*^2^ or *t*-test	*p*-values FDR corrected	Cohen’s *d*
*N* (girls)	16 (6)	16 (11)	3.14	0.08	
Age [years]	10.1 (1.24)	9.90 (1.29)	0.36	0.78	0.13
Min–max	8.34–12.2	8.00–12.3			
Non-verbal intelligence [score]	34.7 (1.35)	32.7 (3.24)	2.28	0.30	0.81
Self-report anxiety [score]	10.1 (5.17)	13.5 (5.18)	−1.88	0.30	−0.66
Working memory [score]	11.3 (2.77)	10.3 (1.44)	1.28	0.53	0.45
Mathematical skills [score]	56.1 (16.4)	51.6 (20.0)	0.69	0.69	0.24
Family SES [score]	7.19 (0.70)	6.50 (1.40)	1.75	0.30	0.62
Parent-report math affect [au]	34.4 (16.7)	30.2 (15.5)	0.73	0.69	0.26
Home Phys. environment [au]	25.63 (4.56)	26.50 (3.61)	−0.60	0.69	−0.21
Parents’ life stress [%]	45.81 (23.41)	54.50 (19.95)	−1.13	0.54	−0.40
Home Ped. environment [au]	7.13 (1.31)	7.25 (1.24)	−0.28	0.78	−0.10

*Note*. Mean and SD in parentheses for all variables except sample size and age range.

*Au* arbitrary unit, *Phys.* physical, *Ped.* pedagogical.

We hypothesized that both groups of children would show neural responses to their self-generated incorrect versus correct responses, and that this neural activity and connectivity would differ in children from these two types of schools. Specifically, we hypothesized that (1) regardless of pedagogical experience, students would show increased activity along the cingulate gyrus to incorrect versus correct answers, consistent with data from adults; and (2) Montessori and traditionally-schooled students would show relatively different patterns of brain activation and connectivity in trials corresponding to correct and incorrect responses: Montessori students would show higher brain activation and connectivity in brain regions implied in error monitoring (ACC, medial frontal cortex), whereas traditionally-schooled students would show effects in brain regions involved in memory (e.g., hippocampus).

## Results

### Group variables

No significant differences were found (*p* > 0.3) for age, fluid intelligence, trait anxiety, working memory, mathematical competency, or affect toward math, revealing comparable groups on these measures; parents’ SES, parenting style, perceived life stress, and pedagogical approach at home did not differ between the groups (Table 1). There were marginally more girls in the traditionally-schooled group (*p* = 0.08). We thus examined the effect of gender and found it not significant (*p* = 0.18).

### Behavioral analysis

**Fig. 1:**
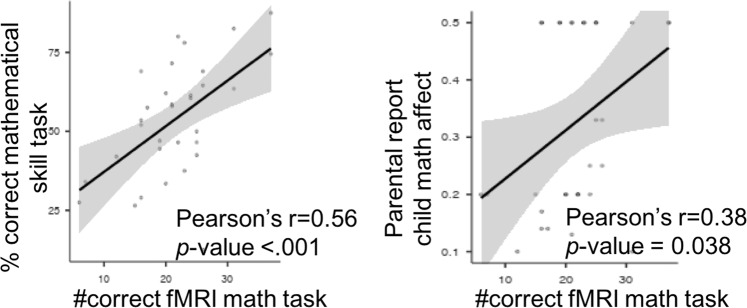
Relations between fMRI math task performance and standardized measures. Participants’ performance on the fMRI math task correlated with their mathematical skills score (standardized task) and with parental report of child’s math affect.

All participants performed at above 60% accuracy and no ceiling effect was observed, suggesting that the fMRI math task’s difficulty level was calibrated well for this age. Validating the fMRI experimental task, as shown in Fig. 1, performance was positively correlated with performance on the standardized math task completed outside the scanner (*r* = 0.56, *n* = 32, *p* < 0.001) and with parental report of the child’s math affect (*r* = 0.38, *n* = 32, *p* = 0.038).

**Fig. 2:**
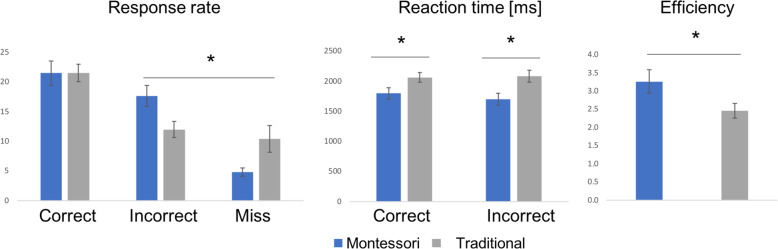
Group averages for behavioral results; response-rate, reaction time and efficiency (computed as the ratio of reaction time to percentage correct responses). Error bars represent SEM.

As expected, participant’s correct responses were more frequent than incorrect or missed (no response) responses, *F*(60,2) = 22.88, *p* < 0.001, *ɳ*_p_^2^ = 0.43. There was an interaction by group in response patterns, *F*(60,2) = 3.78, *p* = 0.028, *ɳ*_p_^2^ = 0.11. While the groups did not differ on rate of correct responses, *t*(60) = −0.03, *p*_tukey_ = 1.0, Montessori participants had higher incorrect rates, while traditionally-schooled students missed more trials, *t*(60) = 3.05, *p*_tukey_ = 0.038 (see Fig. 2, left).

We checked whether right and wrong math problems (in the stimulus presentation) interacted with students’ correct and incorrect responses and found no interaction (no differences in response patterns when presented with right and wrong math problems, *F*(30,1) = 1.46, *p* = 0.237, *ɳ*_p_^2^ = 0.05). There was also no interaction by pedagogical group (*F*(60,2) = 0.23, *p* = 0.638, *ɳ*_p_^2^ = 0.001). However, we did not have sufficient trials to power a test for condition-specific (right/wrong problem presented) response effects at the neural activation level.

Reaction time (RT) was on average faster in Montessori students (*M*_RT_M_ = 1719 ms, SD = 405) than in traditionally-schooled students (*M*_RT_T_ = 2060, SD = 351), *F*(30,1) = 6.55, *p* = 0.016, *ɳ*_p_^2^ = 0.18. RT did not differ according to the response type (correct, incorrect), *F*(30,1) = 0.94, *p* = 0.34, *ɳ*_p_^2^ = 0.03, and no interaction between response type and pedagogical group was found, *F*(30,1) = 2.06, *p* = 0.161, *ɳ*_p_^2^ = 0.06 (see Fig. 2, center).

Overall, efficiency (RT/Accuracy) was higher in Montessori students than in traditionally-schooled students; *M*_M_ = 3.26, SD = 1.31; *M*_T_ = 2.46, SD = 0.81; *t*(30) = 2.09, *p* = 0.046, Cohen’s *d* = 0.74 (see Fig. 2, right).

### Neural activation analyses

**Fig. 3:**
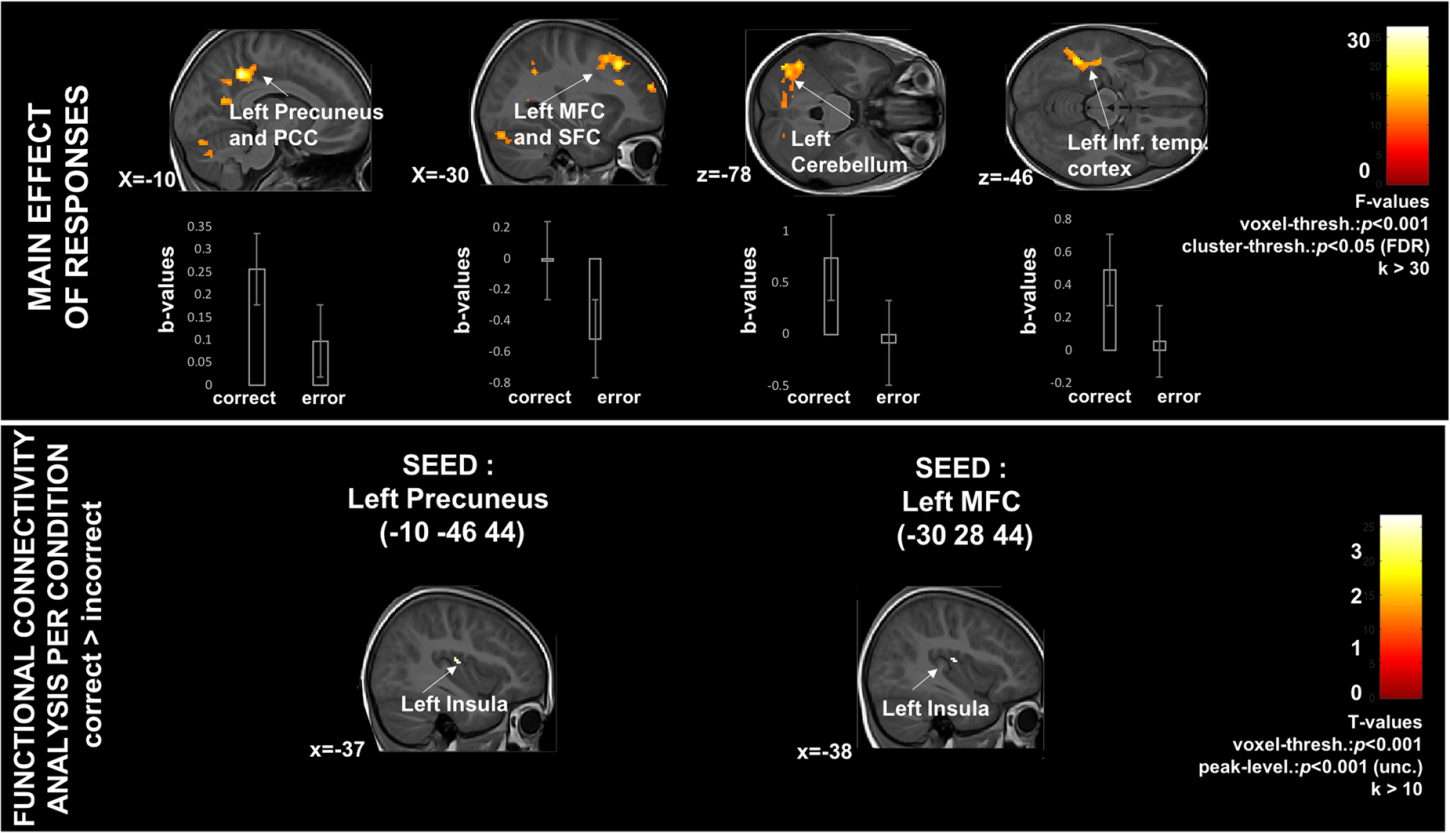
Relative to erroneous responses, correct responses elicited higher brain activity and functional connectivity across pedagogical groups. PCC posterior cingulate cortex, MFC medial frontal cortex, SFC superior frontal cortex. Error bars represent SEM.

**Fig. 4:**
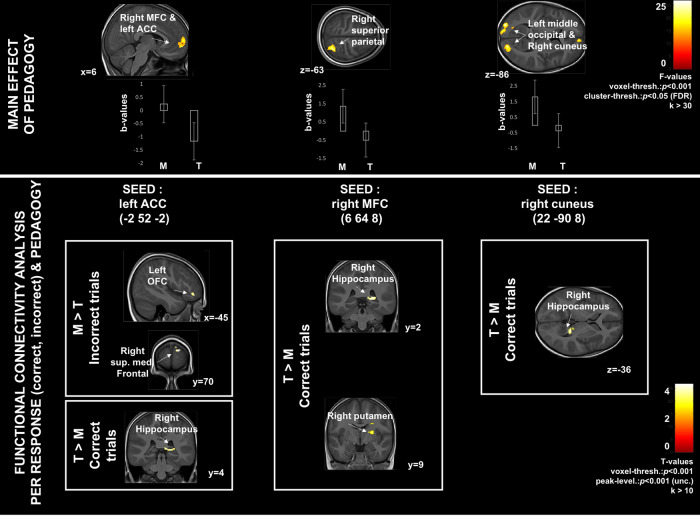
Effect of pedagogy on neural activations and functional connectivity during the math task. Relative activation was higher in Montessori students (M) compared with traditionally-schooled students (T). Functional connectivity analyses by pedagogical group for trials with correct versus incorrect responses: Montessori students (M) showed stronger connectivity (higher correlation) with seed regions for incorrect responses, while traditionally-schooled students (T) showed stronger connectivity (higher correlation) for correct responses. MFC medial frontal cortex, ACC anterior cingulate cortex, OFC orbitofrontal cortex. Error bars represent SEM.

The two-way mixed-design ANOVA with response (student is correct or incorrect) as within-subject factor and pedagogy (Montessori, traditional) as between-subjects factor revealed main effects of both factors, but no interaction. First, relative to incorrect responses, correct responses elicited higher brain activity (Fig. 3 top panel, Table 2A) in the bilateral posterior cingulate cortex and left precuneus, as well as within the middle frontal gyrus, the left inferior temporal gyrus, and both left 1st and 2nd crus of the cerebellum. Second, regardless of whether the response was correct or incorrect, relative to the traditionally-schooled students, the Montessori students showed increased activation of the left occipital cortex and right cuneus (Fig. 4 top panel, and Table 2B), and contralateral regions at trend-level; the right superior parietal lobule; and the medial prefrontal cortex (with some activation extending into the left anterior cingulate cortex).

**Table 2:** Cluster-level information corresponding to the ANOVA fMRI activation results shown in Figs. 4 and 5, top panels.

MNI coordinate of local peak in (*x*, *y*, *z*)	Peak location according to automated anatomical labeling (aal)	*Z*-value	Cluster extent (in mm^3^)
(A) Main effect of response—voxel threshold at *p* < 0.001 and cluster threshold at *p* < 0.05 (FDR)			
−10, −46, 44	Left precuneus	4.76, *p* < 0.000	1306
−2, −42, 32 4, −48, 30	Left PCC Right PCC		
−30, 28, 44	Left MFC	4.43, *p* < 0.000	569
−44, −78, −30 −4, −80, −22	Left crus1 cerebellum Left crus2 cerebellum	4.17, *p* < 0.000	693
−44, −46, −14	Left inferior temporal cortex	4.09, *p* = 0.002	372
(B) Main effect of pedagogy			
20, −92, 10	Right cuneus	4.41, *p* = 0.006	300
−28, −96, 10	Left middle occipital	4.31, *p* < 0.000	703
30, −56, 66	Right superior parietal	4.09, *p* = 0.010	270
6, 64, 6 −2, 52, −2	Right. MFC Left ACC	4.08, *p* = 0.019	234
(C) Interaction term			
	No		

*PCC* posterior cingulate cortex, *MFC* medial frontal cortex, *ACC* anterior cingulate cortex.

We excluded the miss condition from the current analysis as a skipped response could be due to a range of situations, from fatigue to delayed button press to loss of concentration or uncertainty about the answer. Future research should differentiate brain activity underlying trials “missed” for these various reasons, especially given the group difference in frequency of missed responses.

### Functional connectivity analyses

The functional connectivity analyses revealed that connectivity differed depending on whether students responded correctly or incorrectly. Independent of pedagogical group, the precuneus and the mid-frontal cortex seeds were more strongly connected to the left insula for correct responses compared with incorrect responses (Fig. 3 bottom panel, Table 3A). Connectivity patterns in correct compared with incorrect responses also differed by pedagogical group. For correct responses, the traditional pedagogy group showed stronger connectivity (higher correlation) between each seed (the left anterior cingulate cortex, the right medial prefrontal cortex, and the right cuneus) and the right hippocampus. Traditionally-schooled students also showed stronger connectivity during correct trials between the right medial prefrontal cortex seed and the right putamen. There were no regions that showed greater connectivity for correct responses for the Montessori group. For incorrect responses, Montessori students showed greater connectivity between the left anterior cingulate cortex seed and the right middle and superior frontal regions, and the left orbitofrontal cortex (Fig. 4 bottom panel, Table 3B). There were no regions that showed greater connectivity for incorrect responses for the traditionally-schooled group.

**Table 3:** Peak-level information corresponding to the functional connectivity (PPI) results shown in Figs. 3 and 4.

SEEDS	Condition	MNI coordinate of peak in (*x*,*y*,*z*)	Peak location according to automated anatomical labeling (aal)	*T*-value
(A) Functional connectivity from the SEEDs selected from main effect of correct vs incorrect response—voxel threshold at *p* < 0.001 and peak-level threshold at *p* < 0.001 (uncorr.)				
Left MFC	Correct > Incorrect Incorrect > Correct	−38, −16, 16	Left insula	3.77, *p*_unc._ < 0.001
Left precuneus	Correct > Incorrect Incorrect > Correct	−38, −18, 16	Left insula	3.72, *p*_unc._ < 0.001
(B) Functional connectivity from the SEEDs selected from main effect of pedagogical group				
Left ACC	M > T correct responses			
	T > M correct responses	24, −38, 4 34, 60, 28	Right hippocampus Right MFC	4.44, *p*_unc._ < 0.001 3.70, *p*_unc._ < 0.001
	M > T incorrect responses	14, 68, 26 −44, 42, −8	Right SFC Left inf. OFC	3.17, *p*_unc._ = 0.001 3.26, *p*_unc._ = 0.001
	T > M incorrect responses			
Right MFC	M > T correct responses			
	T > M correct responses	24, −38, 4 26, −6, 10	Right hippocampus Right putamen	4.53, *p*_unc._ < 0.001 4.04, *p*_unc._ < 0.001
	M > T incorrect responses			
	T > M incorrect responses			
Right cuneus	M > T correct responses			
	T > M correct responses	24, −38, 4	Right hippocampus	4.62, *p*_unc_. < 0.001
	M > T incorrect responses			
	T > M incorrect responses			

*MFC* medial frontal cortex, *ACC* anterior cingulate cortex, *SFC* superior frontal cortex, *OFC* orbitofrontal cortex.

## Discussion

Though error monitoring is fundamental to learning and develops across childhood and adolescence, to our knowledge, few studies have examined its neural correlates in children^13,14,42^ and no study has examined its susceptibility to pedagogical approaches. Accordingly, the goal of this study was to identify brain activity and functional connectivity during math-task error monitoring in 8–12-year-old schoolchildren from Montessori and traditional schools. Interestingly, we found that the schoolchildren in our samples showed greater neural activity for their correct than for their incorrect responses in various regions, independent of pedagogical experience. This was found in several regions, including regions implicated in the default mode and executive networks. This runs against previous studies with adults^8,43–46^, which report greater activity for incorrect responses.

We also found that pedagogical exposure was associated with the behavioral and neural correlates of error monitoring in our samples. Even with similar levels of math competency to traditionally-schooled students, and similar proportions of correct answers in our task, we found that Montessori students reacted more quickly during the task and made more incorrect responses but missed fewer trials compared with traditionally-schooled students. Across conditions, Montessori participants showed higher brain activity than traditionally-schooled students in regions implicated in visual and math processing, as well as in regions related to attentional/executive control.

Most interestingly, the groups’ functional connectivity patterns following their correct and incorrect responses differed. We used the neural activity contrast of correct versus incorrect participants’ responses to identify seed regions in the ACC, the cuneus cortex and the right superior medial frontal cortex. These regions are interesting for our research question because they have been shown to be involved in self-monitoring^11,47^. Montessori students showed stronger connectivity (higher correlation) between these seed regions and the ventromedial prefrontal cortex in trials in which they had made errors. By contrast, traditionally-schooled students showed stronger connections between the seed regions and the hippocampus on trials in which they had answered correctly. Montessori students did not show significant changes in connectivity following correct responses, and traditionally-schooled students did not show significant changes following incorrect responses.

Both the Montessori and traditionally-schooled groups showed greater neural activation during correct responses relative to incorrect responses in the precuneus, PCC, MFC, inferior temporal cortex, and cerebellum. This is the opposite pattern than has generally been observed in adults^7^, and deserves additional experimental attention in future work on the development of error monitoring in children. One possible interpretation comes from the observation that our findings align with earlier work showing a stronger impact of positive than negative feedback on learning in late childhood^48^. This difference between adults’ and children’s responses to feedback has been proposed to reflect a change in the information that children find salient for learning, rather than a change in their perception of the affective value of the feedback^49^. Though further work is needed, it is possible that our neural finding corresponds to a developmental difference between children and adults around the saliency and utility of correct responses. It could be that prior to reaching a level of competence in math that allows for reliable error prediction, children may rely more heavily on associative learning and on integrating into their knowledge schemas the procedures that led them to correct information. This would make correct responses more relevant for learning and therefore more neuropsychologically salient. At the same time, an experience-dependent shift in subsequent processing, given the known network plasticity of error monitoring in children, could still be possible and would be reflected in pedagogical group differences in connectivity. A heavier reliance on integration of procedures leading to correct responses would also be consistent with the increased PCC and cerebellar activations that we observed to correct responses in both pedagogical groups. PCC is a highly anatomically connected hub central to the default mode network^50^, and is involved in the switch from internally to externally directed attention^51,52^, as well as in forming integrated memories^52–54^. The activated sectors of cerebellum are involved in many cognitive functions involving associative and procedural learning^55^.

Our findings open the question of whether these developmental processes are specific to math learning, or reflect the development of more general attentional and learning mechanisms. Here, the increase in activity in the ventral anterior sector of the precuneus^56,57^, left middle frontal gyrus^58^, and Crus 1 and Crus 2 of the cerebellum^55,59,60^, could reflect greater executive control and external focus when responding correctly, and integration of executive control with regions known to be involved in mathematical cognition, including inferior temporal gyrus and the lateral frontal area^61^. Whether the student would be correct as a consequence of his or her cognitive control and engagement of mathematical processing regions, or whether being correct would elicit higher engagement, is a topic for future work.

The finding that Montessori students missed fewer trials and had more incorrect trials could reflect the emphasis on exploratory learning in Montessori classrooms^62,63^. The extent of exploratory learning through trial-and-error is known to depend on the structure of the environment, the task complexity and the instructions given; these features together have been shown to impact self-directed executive functions and curiosity among children^64–67^. This explanation would also be consistent with the fact that, in our study, Montessori students’ showed stronger neural activation during math processing in bilateral occipital and parietal cortices, involved in multisensory integration^68,69^, and in the right inferior parietal lobule, known to be recruited for math processing^70^. Montessori students also showed increased connectivity during incorrect trials with frontal areas. One testable hypothesis for future work is that these patterns of results in Montessori students reflect a more exploratory, self-corrective and multisensory approach to mathematical cognitive processes. Conversely, it may be that the traditionally-schooled children’s increased functional connectivity between the ACC and the hippocampus reflects a strategic inclination to either memorize or recall correct answers, consistent with instrumental learning^71^ and/or reinforcement learning^72^, with less reliance on self-direction and self-monitoring of errors. Together, these results suggest that daily pedagogical experience may have important implications for learning, behavior and related mindsets (i.e., being more oriented toward processes versus outcomes^73^) that should be further explored.

There are a few limitations to be mentioned. First, our study compared groups that were not randomly assigned to either Montessori or traditional education, making it possible that, despite our efforts to control for relevant variables, the observed effects were also driven by other factors (e.g., family-related or motivation-related) than the pedagogical ones. Second, our study has a modest sample size and a cross-sectional design. While our results suggest that error monitoring is modulated by pedagogical experience, further longitudinal and larger-scale studies will need to investigate the extent to which pedagogy contributes to the emergence of robust psychological and neural error processing. Such studies would help to further probe the role of context in the development of error monitoring for both Montessori and traditionally-schooled students, and could inform pedagogical and policy-related decisions that aim to foster process-oriented learning behaviors^74^. Another limitation of our study is that it did not include adult participants so we could not test whether brain patterns to correct and incorrect responses in our specific task are development-related or persist in adulthood. To confirm brain activation differences are development-related, it would be of interest to have adults perform the same experiment (with tracking of their pedagogical history).

To conclude, our findings suggest that 8–12-year-old students may process correct and incorrect responses differently than do adults, and that they may attend especially to correct responses. Our findings also suggest that pedagogical experience in school modulates error-monitoring behavior and its underlying brain activity and connectivity. Together, these findings call for further research testing whether error-monitoring competencies and corresponding brain networks indeed undergo a shift with age that is modulated by pedagogical experience.

## Methods

### Participants

Thirty-seven healthy children (18 females; aged 8–12.3 years, mean ± SD = 9.95 ± 1.25) completed the experiment as part of a larger study including neuroimaging and behavioral measures aimed at evaluating the impact of school environment on cognitive and emotional development of error monitoring. Selection criteria were age (8–12 years of age) and school enrollment (participants had to be enrolled in a Montessori or in a traditional school system from Kindergarten on, or for at least 3 years in the case of the youngest children). All but one participant in each group were right-handed. One Montessori participant stopped the task half way due to sickness in the scanner, and four others were excluded due to dental braces that would interfere with the fMRI scan (*n* = 1 traditional student), high dyslexia and dyscalculia (*n* = 1 Montessori student), or motion >3 mm exceeding a rate of 20% of the slices collected (*n* = 2, one from each group), leaving 32 subjects (17 female; aged 8–12.3 years, mean ± SD = 9.98 ± 1.25) available for analyses (half from each schooling system). This study was approved by the local ethics committee (CER-Vaud). Written informed consent to take part in the study was obtained from parents and oral assent from subjects; participants acknowledged that they were free to withdraw at any time without penalty. Participants were compensated with a voucher and received the personalized gift that had been displayed during the fMRI task.

### Group variables

Data used to evaluate between-group homogeneity were:

(i) Non-verbal intelligence (black and white version of the Progressive Matrices^75^); the child had to choose from amongst six items one pattern that would fit within a matrix. There were 36 matrices to be completed and each correct answer granted a point (maximum 36).
(ii) Self-reported anxiety (STAI-Y2^76^); on a 3-point Likert scale, the child responded to questions about their state anxiety. Responses were summed (scores can range from 0 to 40; higher scores denote higher anxiety).
(iii) Working memory (digit–letter span tasks^77^); the child listened to and memorized a string of mixed digits-letters and repeated them in ascending order (that is, they mentally reorganized the information). The score was age-standardized (higher scores denote greater working memory capacity).
(iv) Mathematical skills^78^; the child solved standardized math problems including arithmetic, logic, and geometric paper-based tasks. The maximum score was 100% correct answers.

Finally, to control as best as possible for the possibility of selection bias stemming from recruiting Montessori students from private schools and traditional students from public schools, to ensure home environments were similar, and to ensure equivalence on math anxiety (which is known to be impacted by parents^79^) we measured:

(i) Family’s socio-economic status, including both parents’ education levels and current job (higher scores denote higher socio-economic status).
(ii) Parental report of child math affect (parents’ reports of their child’s level of math anxiety and math enjoyment) using a 5-point Likert scale (higher scores denote more positive affect toward math).
(iii) Home physical environment, using a questionnaire about whether there is a yard, the number of rooms, etc. (higher scores denote more enriched home environment).
(iv) Parents’ perceived life stress (higher scores denote higher perceived life stress);
(v) Home pedagogical environment, including questions about parents’ interest in education and pedagogy (e.g., how many books on education they have at home), and style of parenting (e.g., number of meals shared with the child per week on average, frequency of museum visits together, type of feedback given when the child succeeds); (higher scores denote increased knowledge about pedagogy and more parental involvement in their child’s intellectual development).

Control variables were partially collected online (parental questionnaires; children’s fluid intelligence and self-reported anxiety) and through a behavioral assessment that took place after fMRI scanning.

A chi-square test was performed to determine whether the gender ratios differed between Montessori and traditional students. In addition, multiple *t*-tests (independent or Welch’s according to the preliminary data check with *Q*–*Q* plots and Levene’s test) with a 95% confidence interval (CI) for the mean difference were run on the control variables to test for significant differences between the groups (Montessori vs traditional), with a false-rate discovery (FDR) *p*-value correction at *q* = 0.05.

### Task and procedure

**Fig. 5:**
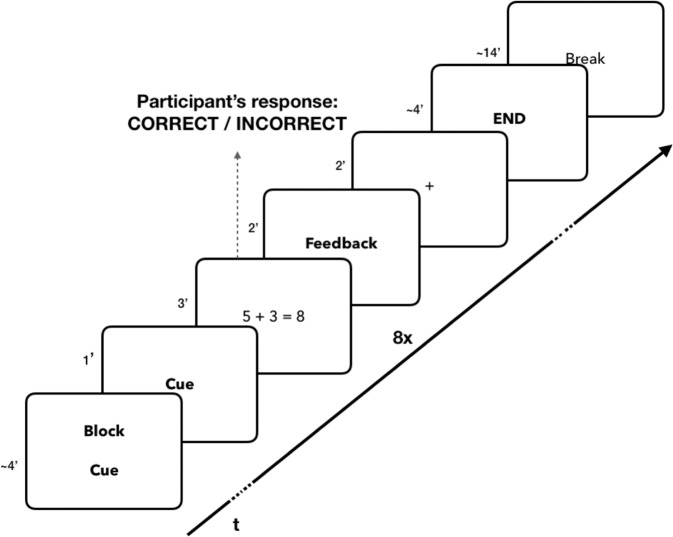
The fMRI task design: after the cue presentation (e.g., “If you perform well, you will gain points”), the participant had to determine whether the math problem and its suggested solution (e.g., “3 + 10 = 12”) was right or wrong. Feedback was given, based on his/her real performance.

Students were individually assessed on a novel math proofreading fMRI paradigm that was designed to evoke a school-related task. During scanning, participants were asked to respond as to whether the solution of the math problem they viewed was right or wrong using a response box in their right hand. Instructions emphasized the need for both speed and accuracy. Each trial consisted of (i) a start cue displayed for 1000 ms, followed by (ii) a simple addition or subtraction problem with a suggested solution that could be correct or incorrect (retrieved from a standardized age-normalized task, presented in random order^80^; displayed for 3000 ms, during which the participant had to respond, (iii) the feedback (words “correct” or “incorrect”) displayed for 2000 ms, and (iv) a fixation cross as inter-trial jitter lasting between 2000–3000 ms, in steps of 500 ms, varying randomly to provide adequate temporal sampling of the blood oxygen-level dependent (BOLD) response (Fig. 5). In total, 64 trials were divided evenly into eight blocks with an inter-block interval of 14,000 ms.

Stimulus delivery and recording of behavioral data (reaction time and accuracy) were controlled by E-prime and a serial response box (www.pstnet.com; Psychology Software Tools). Button presses occurring more than 3000 ms after stimulus the presentation of the math problem were labeled as a miss, and were excluded from neuroimaging analysis.

The data were collected as part of a larger study examining the differential effects of various rewards versus no reward or no feedback on correct versus incorrect responses. Because of the short experiment duration necessary for child participants, we were unable to include sufficient trials within each reward condition to test condition-specific reward effects on error monitoring. The current study therefore focused exclusively on the comparison between “correct versus incorrect” trials. To avoid confounding with reward, reward images were presented concurrent with the feedback for both correct and incorrect trials.

### Data acquisition

Structural and functional images were collected at the Lemanic Biomedical Imaging Center (CIBM) of the University Hospital Lausanne (CHUV), on a Siemens 3T Prisma-Fit MR scanner, with a 64-channel head-coil. For each participant, a 3-dimensional high-resolution isotropic T1-weighted sequence (MPRAGE) was acquired (TR = 2000 ms, TE = 2.47 ms, 208 slices; voxel size = 1 × 1 × 1, flip angle = 8°) as anatomical individual reference and basis for surface reconstruction. The functional scans were continuously acquired using a standard echo-planar gradient echo sequence acquired by simultaneous multislice (SMS) imaging technique and covering the whole brain with an isotropic voxel size of 2 mm ([TR] = 1000 ms; echo time [TE] = 30 ms; 64 axial slices; slice thickness = 2 mm, no gap between slices, flip angle = 80°, matrix size = 100 × 100, field of view [FOV] = 200 mm, sms factor = 4, parallel imaging acceleration factor = 2). For each subject one session of 740 volumes was recorded, including seven “dummy” scans that were then discarded by the scanner, for a total acquisition time of 12 min and 26 s. Foam pads were placed around the subject’s head inside the coil to prevent head motion.

### Behavioral analysis

Behavioral data were analyzed using the statistical R software jamovi (Jamovi Project, 2018). First, to validate the fMRI task, Pearson correlation coefficients were computed to assess the relationship between correct response scores and the participant’s standardized math-task performance and parental report of affect toward math. Second, main effects and their interaction on accuracy were analyzed using a 3-by-2 ANOVA (response type -correct, incorrect, missed-, as within-subject factor; Montessori versus traditional as between-subject factor). Main effects and their interaction on response time were analyzed using a 2-by-2 ANOVA (response type -correct, incorrect-, as within-subject factor; Montessori versus traditional as between-subject factor), with *α* < 0.05. Post hoc Tukey tests were computed when relevant. Finally, we computed participants’ efficiency as their reaction time divided by their proportion of correct responses^81^ and an independent *t*-test was used to statistically evaluate group differences (Montessori versus traditional students).

### Neural activation analyses

Imaging data processing and analyses were carried out with Matlab (Mathworks, Natick, MA, USA Version 7.13) using the software SPM12 (Wellcome Department of Cognitive Neurology, London, UK) and the results were visualized using xjview Toolbox for SPM (http://www.alivelearn.net/xjview) and MRIcroGL (http://www.cabiatl.com/mricrogl/). Anatomical locations were labeled and described with the help of the aal atlas.

Functional images were motion corrected with reference to the first scan, using a 6-parameter rigid-body realignment. Then, slice timing correction was performed on these realigned images. The functional images were then co-registered to the high-resolution T1 anatomical image of the participant, using mutual information. Finally, images were normalized (by estimation based on the anatomical images and then applied to the functional images) to the MNI template and spatially smoothed using an 8-mm Gaussian filter. Visual inspection of estimated motion parameters was conducted on a subject-by-subject basis, and subjects demonstrating a rate of motion-corrupted scans (>3 mm, >3°) exceeding 20% were excluded (*n* = 2).

First-level statistics were performed for each subject using a general linear model as implemented in SPM12. Brain activity of interest comprised the 4-s stimulus presentation that followed the start cue, including task and feedback time. Contrasts of the participant’s correct vs. incorrect responses were computed. The realignment parameters were included in the model as a nuisance variable, and the highpass filter cut-off was set to 128 s. The generated maps were then used as input values for the group-level analysis. Second-level random effects were analyzed using general mixed-design ANOVA including the factors Response (participant’s correct or incorrect responses) as within-subjects factor, and Pedagogy (Montessori, traditional) as between-subjects factor. All activation maps were thresholded at *p* < 0.05 for cluster-level FDR correction, which correspond to a voxel-wise threshold of *p* < 0.001 and a cluster size threshold of >30 voxels per cluster.

To investigate effects of response type and pedagogical group on functional connectivity, selected seed regions of interest (ROIs) identified in the activation analysis were used as seeds in a psycho-physiological interaction (PPI) analysis implemented in SPM12. ROIs in the middle prefrontal cortex (*x* = −30, *y* = 28, *z* = 44) and ventral anterior precuneus (*x* = −10, *y* = −46, z = 46) were identified from the main effect of correct versus incorrect responses; ROIs in the right prefrontal cortex (*x* = 6, *y* = 64, *z* = 8), anterior middle cingulate (*x* = −2, *y* = 52, *z* = −2), and cuneus (*x* = 22, *y* = −90, *z* = 8) were identified from the main effect of pedagogical group. Seeds were defined as 8-mm-radius spheres. The average connectivity maps were computed for each subject by response type (correct and incorrect). We conducted a second-level group analysis using a *t*-test for each response type (correct versus incorrect), or pedagogical group (Montessori versus traditional). As the connectivity analyses were exploratory, the threshold was set at *p* < 0.001 uncorrected at the voxel-wise level.

## Supplementary information

nr-reporting-summary

## Data Availability

Archives of behavioral data used in this study, including the e-Prime stimulus files, can be accessed in Zenodo 10.5281/zenodo.3773305.

## Code availability

Imaging data preprocessing was carried out with Matlab (Mathworks, Natick, MA, USA, Version 7.13) using the software SPM12 (Wellcome Department of Cognitive Neurology, London, UK). The preprocessing pipeline that support the findings of this study is available from the corresponding author upon request.
